# Nomogram Predicts the Role of Primary Tumor Surgery on *De Novo* Stage-IV Breast Cancer Patients: A SEER-Based Competing Risk Analysis Model

**DOI:** 10.3389/fonc.2022.819531

**Published:** 2022-05-04

**Authors:** Hanxiao Cui, Luyao Dai, Yuanhang Bao, Liqun Hu, Zhangjian Zhou, Meng Wang, Shuai Lin, Hao Wu, Xiaobin Ma, Huafeng Kang

**Affiliations:** ^1^Department of Oncology, The Second Affiliated Hospital, Xi’an Jiaotong University, Xi’an, China; ^2^School of Basic Medical Sciences, Xi’an Key Laboratory of Immune Related Diseases, Xi’an Jiaotong University, Xi’an, China

**Keywords:** SEER, *de novo* stage-IV breast cancer, surgery, competing risk model, nomogram, metastatic pattern

## Abstract

**Objective:**

The efficacy of primary tumor surgery on survival in female patients with *de novo* stage IV breast cancer (BC) remains unclear. Our study endeavored to develop comprehensive competing risk nomograms to predict clinical outcomes and guide precision treatment in these patients.

**Participants and Methods:**

A total of 12281 patients who had distant metastasis at initial BC diagnosis between 2010 and 2017 in the Surveillance Epidemiology and End Results (SEER) database, were enrolled in this study. First, we assessed the impacts of primary tumor surgery on overall survival (OS) and breast cancer-specific survival (BCSS) using the Kaplan-Meier curves. Then subgroup analyses stratified by different metastatic patterns were performed using Cox and competing risk models (CRM). Based on the filtered independent prognostic parameters by CRM, we established two nomograms to predict the probability of breast cancer-specific death (BCSD) at 1-,2- and 3-year intervals. Furthermore, calibration curves and area under the curves (AUC) were conducted for validation.

**Results:**

Kaplan-Meier analysis revealed that surgery was associated with better OS and BCSS (P<0.001). Subgroup analyses demonstrated that in bone-only metastases pattern, relative to breast-conserving surgery (BCS), patients receiving mastectomy had worse prognosis and the poorest survival belonged to non-surgery individuals (BCSS: mastectomy: HR=1.35; 95%CI=1.15-1.60; non-surgery: 2.42; 2.08-2.82; OS: mastectomy: 1.44; 1.23-1.68; non-surgery: 2.40; 2.08-2.78). Additionally, no survival difference was observed between BCS and reconstruction recipients (BCSS: HR=1.10; 95%CI=0.85-1.43; OS: 1.11; 0.86-1.44). Furthermore, patients undergoing BCS possessed similar BCSS with mastectomy recipients as well as reconstruction recipients in viscera metastases pattern, whereas non-surgery individuals had a worse survival (mastectomy: HR=1.04; 95%CI=0.92-1.18; reconstruction: 0.86; 0.69-1.06; non-surgery: 1.83; 1.63-2.05). Two competing risk nomograms of distinct metastatic patterns were established to comprehensively predict the survival of patients. Calibration curves indicated the terrific consistency of the models. Moreover, the AUC values in the training and validation sets were in the range of 0.70–0.80, exhibiting good specificity and sensitivity.

**Conclusion:**

The surgery implementation was associated with a lower probability of BCSD in *de novo* stage-IV BC patients. Our nomograms could offer a relatively accurate and individualized prediction of the cumulative incidence rate of BCSD after primary tumor resection.

## Introduction

According to the most recent report from the International Agency for Research on Cancer (IARC), new cases of breast cancer (BC) rapidly grew to 2.26 million in 2020. Besides, it has officially overtaken lung cancer as the major component of malignant tumors worldwide and maintained the leading cause of cancer-related death in females (1, 2). Approximately 5-8% of BC patients exhibit distant metastases at initial diagnosis (3). In addition, stage-IV BC is considered to be incurable with a relatively short median OS despite tremendous advances in systemic therapeutics. In view of these unfavorable prognoses, the chief objective of treatment is to mitigate symptoms, improve the quality of life and ameliorate survival (4, 5). It is generally accepted that systemic therapeutics, including chemo, endocrine, and targeted therapy, are the fundamental and effective treatments for MBC (6). However, due to the lack of consensus, the essential role of primary tumor resection in MBC patients is still controversial.

A multitude of retrospective studies has demonstrated that surgical resection of primary tumors extended the life expectancy of MBC patients (7–12). Nevertheless, four prospective randomized trials showed contentious results (13–16). MF07-01 trial was the only trial that observed survival benefits from locoregional surgery, with a remarkable improvement of 5-year OS (13). However, no statistical differences were found between primary tumor surgery and prognosis in the other trials (NCT00193778, ABCSG-28 POSYTIVE, and ECOG ACRIN 2018) (14–16). The discrepancy in outcomes may be ascribed to different metastatic patterns (13, 17). Furthermore, we are awaiting the results of several well-designed prospective trials, which are still following-up.

In our study, we meticulously probed the effectiveness of locoregional surgery in different metastatic patterns among *de novo* stage-IV BC patients using data from the Surveillance, Epidemiology, and End Results (SEER) database. Moreover, two nomograms considering competitive events were established, making up for a few limitations of retrospective study and offering precise prediction of survival outcomes.

## Methods

### Patient Selection

The following inclusion criteria were used: 1) primary BC and 2) stage IV BC diagnosed from 2010 to 2017.The exclusion criteria comprised of the following: 1) not diagnosed by histology, 2) with unknown metastatic status and surgery data, 3) diagnosed by autopsy and death certificate, 4) with incomplete survival data, 5) without complete clinicopathological data, and 6) male patients.

**Figure 1:**
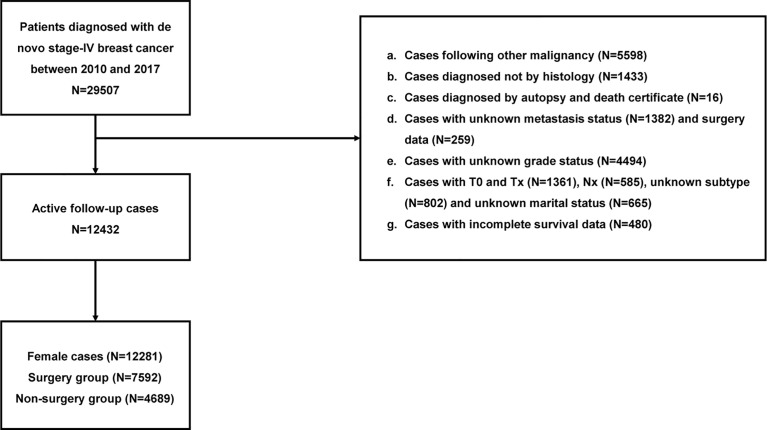
Flow chart of patient selection.

Of the 12281 individuals in our study, 4689 cases undergoing primary tumor surgery were subdivided into the BCS, mastectomy, and reconstruction groups. Additionally, age at diagnosis, year of diagnosis, race, marriage, grade, histology, tumor size, lymph nodes status, subtype, metastatic pattern, radiation, chemotherapy, surgery information, and survival data were obtained from the database. Detailed information is exhibited in **Figure 1**.

### Ethics Statement

The SEER database was set up by the National Cancer Institute of America, which covers approximately 30% of the U.S. population. We signed an agreement to access the SEER research data for this study. In addition, as the database is publicly available, our study was exempt from the ethical board of The Second Affiliated Hospital of Xi’an Jiaotong University.

### Endpoints

The follow-up period ended in November 2020 and the median follow-up time was 25 months (1-107 months). In addition, the primary endpoints of this study were BCSS and BCSD, which were defined as the interval from diagnosis to death due to BC. The subordinate outcome was OS, which referred to the interval from BC diagnosis to death of any cause.

### Statistical Analysis

We employed descriptive statistics to analyze the clinicopathological characteristics. Age at diagnosis, year of diagnosis, race, marriage, grade, histology, tumor size, lymph nodes status, subtype, metastatic pattern, radiation, chemotherapy, surgery information, and survival data were selected as variables. We conducted a chi-squared test of these variables to compare the variations between distinct surgical procedures. Kaplan-Meier analysis and log-rank test were completed to plot curves and compare the divergency in OS and BCSS. Subgroup analyses of different metastatic patterns were performed using Cox proportional hazard model and competing risk model to explore the independent prognostic factors. Based on the filtered variables, we constructed two competing risk nomograms to predict the probability of 1-, 2-, and 3-year BCSD. In addition, calibration curves and AUC values were used to assess the reliability of the model. All statistical assays were completed using R software 4.0.3. Statistical significance was defined as a two-sided P-value <0.05.

## Results

### Clinicopathological Characteristics

A total of 12281 patients diagnosed with *de novo* stage-IV BC (2010–2017) were qualified for analyses. Of the 4689 (38.18%) individuals experiencing locoregional surgery, 1379, 2800, and 510 cases were subdivided into BCS, mastectomy, and reconstruction groups respectively, while the remaining 7592 patients (61.82%) avoided surgical interventions. Among these patients, 39.19% were 56-70 years old, 74.16% were white, 52.24% were in single status, 50.68% had poor differentiation (grade III-IV), 76.97% were invasive ductal carcinoma, 35.09% had T4 stage, 47.22% had N1 stage, 58.25% were luminal A subtype, 61.50% had viscera metastases, 63.55% did not receive radiotherapy, and 65.78% underwent chemotherapy. Significant differences were observed in age, race, marriage, grade, histology, T, N, subtype, metastatic status, radiotherapy, and chemotherapy between the four groups (**Table 1**).

**Table 1:** The baseline characteristics of *de novo* stage-IV BC patients with different surgical methods.

	Non-surgery	Mastectomy	Reconstruction	BCS	P
	N (%)	N (%)	N (%)	N (%)	
**Age**					<0.001
<=40	558 (7.35)	281 (10.04)	113 (22.16)	127 (9.21)	
41-55	1961 (25.83)	807 (28.82)	220 (43.14)	420 (30.46)	
56-70	3023 (39.82)	1086 (38.79)	149 (29.22)	555 (40.25)	
>70	2050 (27.00)	626 (22.36)	28 (5.49)	277 (20.09)	
**Race**					0.003
Black	1322 (17.41)	496 (17.71)	69 (13.53)	196 (14.21)	
White	5618 (74.00)	2029 (72.46)	391 (76.67)	1070 (77.59)	
Other	652 (8.59)	275 (9.82)	50 (9.80)	113 (8.19)	
**Marriage**					<0.001
Married	3405 (44.85)	1397 (49.89)	319 (62.55)	744 (53.95)	
Single	4187 (55.15)	1403 (50.11)	191 (37.45)	635 (46.05)	
**Grade**					<0.001
I-II	4099 (53.99)	1124 (40.14)	229 (44.90)	605 (43.87)	
III-IV	3493 (46.01)	1676 (59.86)	281 (55.10)	774 (56.13)	
**Histology**					<0.001
IDC	5837 (76.88)	2105 (75.18)	407 (79.80)	1104 (80.06)	
ILC	731 (9.63)	249 (8.89)	43 (8.43)	116 (8.41)	
Others	1024 (13.49)	446 (15.93)	60 (11.76)	159 (11.53)	
**T**					<0.001
T1	891 (11.74)	179 (6.39)	52 (10.20)	304 (22.04)	
T2	2466 (32.48)	862 (30.79)	208 (40.78)	710 (51.49)	
T3	1379 (18.16)	623 (22.25)	123 (24.12)	175 (12.69)	
T4	2856 (37.62)	1136 (40.57)	127 (24.90)	190 (13.78)	
**N**					<0.001
N0	1745 (22.98)	316 (11.29)	60 (11.76)	380 (27.56)	
N1	4019 (52.94)	1037 (37.04)	195 (38.24)	548 (39.74)	
N2	760 (10.01)	643 (22.96)	119 (23.33)	227 (16.46)	
N3	1068 (14.07)	804 (28.71)	136 (26.67)	224 (16.24)	
**Subtype**					<0.001
HER2-positive	678 (8.93)	312 (11.14)	49 (9.61)	128 (9.28)	
Luminal A	4597 (60.55)	1491 (53.25)	287 (56.27)	779 (56.49)	
Luminal B	1362 (17.94)	482 (17.21)	111 (21.76)	244 (17.69)	
Triple Negative	955 (12.58)	515 (18.39)	63 (12.35)	228 (16.53)	
**Metastatic status**					<0.001
Bone only	2800 (36.88)	1081 (38.61)	239 (46.86)	608 (44.09)	
Viscera	4792 (63.12)	1719 (61.39)	271 (53.14)	771 (55.91)	
**Radiation**					
No/Unknown	5393 (71.04)	1510 (53.93)	232 (45.49)	670 (48.59)	
Yes	2199 (28.96)	1290 (46.07)	278 (54.51)	709 (51.41)	
**Chemotherapy**					<0.001
No/Unknown	2993 (39.42)	705 (25.18)	82 (16.08)	422 (30.60)	
Yes	4599 (60.58)	2095 (74.82)	428 (83.92)	957 (69.40)	

IDC, invasive ductal carcinoma; ILC, invasive lobular carcinoma.

### Impact of Primary Tumor Surgery on Survival in *De Novo* MBC Patients

**Figure 2:**
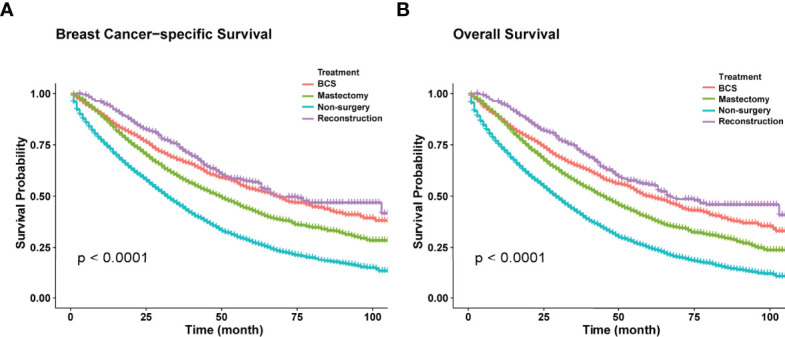
**(A)** Kaplan-Meier curves of BCSS in different surgical methods. **(B)** Kaplan-Meier curves of OS in different surgical methods.

Kaplan-Meier curves showed that surgery improved both OS and BCSS (P<0.001; **Figure 2**). In addition, univariate Cox hazard proportional analysis revealed that age, race, marriage, grade, histology, T, N, subtype, metastatic status, radiotherapy, chemotherapy, and surgical procedures were related to survival (P<0.05; **Table 2**). Considering potential bias, we performed multivariate analysis and confirmed that primary tumor surgery was an independent protective factor for both BCSS (mastectomy: HR, 0.56; 95%CI, 0.52-0.59; BCS: HR, 0.47; 95%CI, 0.43-0.52; reconstruction: HR, 0.45; 95%CI, 0.39-0.52) and OS (mastectomy: HR, 0.56; 95%CI, 0.53-0.60; BCS: HR, 0.49; 95%CI, 0.44-0.53; reconstruction: HR, 0.43; 95%CI: 0.38-0.50).Furthermore, the metastatic pattern was also a crucial independent index that linked to prognosis (**Table 3**).

**Table 2:** Univariate and multivariate Cox hazard proportional model analysis of *de novo* stage-IV BC patients.

	Univariate analysis						Multivariate analysis					
	BCSS			OS			BCSS			OS		
	HR	95%CI	P	HR	95%CI	P	HR	95%CI	P	HR	95%CI	P
**Age**												
<=40	Reference			Reference			Reference			Reference		
41-55	1.20	1.09-1.33	<0.001	1.20	1.09-1.32	<0.001	1.14	1.03-1.27	0.010	1.13	1.03-1.25	0.013
56-70	1.41	1.28-1.55	<0.001	1.46	1.33-1.60	<0.001	1.25	1.13-1.38	<0.001	1.29	1.17-1.42	<0.001
>70	1.94	1.76-2.15	<0.001	2.17	1.97-2.39	<0.001	1.55	1.40-1.72	<0.001	1.70	1.53-1.88	<0.001
**Race**												
Black	Reference			Reference			Reference			Reference		
White	0.70	0.66-0.75	<0.001	0.71	0.67-0.75	<0.001	0.80	0.75-0.86	<0.001	0.80	0.75-0.84	<0.001
Other	0.62	0.56-0.69	<0.001	0.61	0.56-0.68	<0.001	0.72	0.65-0.80	<0.001	0.70	0.64-0.78	<0.001
**Marital status**												
Married	Reference			Reference			Reference			Reference		
Single	1.39	1.33-1.46	<0.001	1.43	1.37-1.50	<0.001	1.21	1.15-1.27	<0.001	1.23	1.17-1.29	<0.001
**Grade**												
I-II	Reference			Reference			Reference			Reference		
III-IV	1.44	1.37-1.51	<0.001	1.38	1.31-1.44	<0.001	1.46	1.38-1.54	<0.001	1.40	1.33-1.48	<0.001
**Histology**												
IDC	Reference			Reference			Reference			Reference		
ILC	0.97	0.89-1.06	0.523	0.97	0.89-1.05	0.462	1.21	1.10-1.32	<0.001	1.16	1.06-1.26	<0.001
Others	1.12	1.04-1.20	0.002	1.12	1.05-1.20	<0.001	1.12	1.05-1.20	0.001	1.12	1.05-1.20	<0.001
**T**												
T1	Reference			Reference			Reference			Reference		
T2	1.06	0.97-1.16	0.184	1.07	0.98-1.16	0.119	1.09	1.00-1.19	0.044	1.10	1.01-1.20	0.022
T3	1.24	1.13-1.37	<0.001	1.22	1.12-1.34	<0.001	1.19	1.08-1.31	<0.001	1.18	1.07-1.29	<0.001
T4	1.58	1.45-1.72	<0.001	1.57	1.44-1.70	<0.001	1.37	1.26-1.49	<0.001	1.35	1.24-1.47	<0.001
**N**												
N0	Reference			Reference						Reference		
N1	0.99	0.93-1.05	0.718	0.96	0.90-1.02	0.141				0.98	0.92-1.04	0.468
N2	0.95	0.87-1.03	0.218	0.91	0.84-0.99	0.024				1.05	0.97-1.15	0.217
N3	1.08	1.00-1.16	0.058	1.03	0.96-1.11	0.427				1.06	0.98-1.15	0.137
**Subtype**												
HER2 Positive	Reference			Reference			Reference			Reference		
Luminal A	1.10	1.01-1.21	0.032	1.10	1.01-1.19	0.030	1.09	0.99-1.20	0.074	1.04	0.95-1.14	0.365
Luminal B	0.80	0.72-0.89	<0.001	0.78	0.70-0.86	<0.001	0.82	0.74-0.91	<0.001	0.78	0.71-0.87	<0.001
Triple Negative	2.87	2.60-3.17	<0.001	2.70	2.46-2.97	<0.001	2.88	2.61-3.19	<0.001	2.68	2.44-2.95	<0.001
**Metastatic status**												
Bone only	Reference			Reference			Reference			Reference		
Viscera	1.47	1.40-1.55	<0.001	1.45	1.38-1.52	<0.001	1.34	1.27-1.42	<0.001	1.33	1.27-1.40	<0.001
**Radiation**												
No/Unknown	Reference			Reference			Reference			Reference		
Yes	0.89	0.84-0.93	<0.001	0.87	0.83-0.91	<0.001	1.11	1.06-1.17	<0.001	1.09	1.03-1.14	0.001
**Chemotherapy**												
No/Unknown	Reference			Reference			Reference			Reference		
Yes	0.72	0.69-0.76	<0.001	0.68	0.65-0.71	<0.001	0.68	0.64-0.72	<0.001	0.66	0.63-0.70	<0.001
**Surgery**												
Non-surgery	Reference			Reference			Reference			Reference		
BCS	0.48	0.44-0.53	<0.001	0.49	0.45-0.53	<0.001	0.47	0.43-0.52	<0.001	0.49	0.44-0.53	<0.001
Mastectomy	0.63	0.59-0.67	<0.001	0.63	0.60-0.67	<0.001	0.56	0.52-0.59	<0.001	0.56	0.53-0.60	<0.001
Reconstruction	0.41	0.35-0.47	<0.001	0.38	0.33-0.44	<0.001	0.45	0.39-0.52	<0.001	0.43	0.38-0.50	<0.001

HR, hazard ratio; CI, confidence interval.

**Table 3:** Univariate analysis of different metastatic patterns.

	BCSS						OS					
	Bone-only			Viscera			Bone-only			Viscera		
	HR	95%CI	P	HR	95%CI	P	HR	95%CI	P	HR	95%CI	P
**Age**												
<=40	Reference			Reference			Reference			Reference		
41-55	1.08	0.91-1.29	0.381	1.28	1.13-1.45	<0.001	1.11	0.93-1.31	0.243	1.26	1.11-1.42	<0.001
56-70	1.38	1.17-1.63	<0.001	1.44	1.28-1.62	<0.001	1.47	1.25-1.72	<0.001	1.47	1.31-1.65	<0.001
>70	1.97	1.66-2.34	<0.001	1.95	1.72-2.21	<0.001	2.25	1.91-2.66	<0.001	2.15	1.91-2.43	<0.001
**Race**												
Black	Reference			Reference			Reference			Reference		
White	0.66	0.59-0.74	<0.001	0.75	0.70-0.81	<0.001	0.66	0.60-0.73	<0.001	0.75	0.70-0.81	<0.001
Other	0.62	0.52-0.75	<0.001	0.62	0.55-0.70	<0.001	0.60	0.50-0.71	<0.001	0.62	0.55-0.70	<0.001
**Marital status**												
Married	Reference			Reference			Reference			Reference		
Single	1.35	1.24-1.46	<0.001	1.41	1.33-1.50	<0.001	1.42	1.31-1.54	<0.001	1.44	1.36-1.52	<0.001
**Grade**												
I-II	Reference			Reference			Reference			Reference		
III-IV	1.42	1.31-1.55	<0.001	1.32	1.24-1.40	<0.001	1.36	1.25-1.47	<0.001	1.27	1.20-1.35	<0.001
**Histology**												
IDC	Reference			Reference			Reference			Reference		
ILC	1.22	1.09-1.37	<0.001	0.94	0.82-1.08	0.376	1.19	1.07-1.33	0.002	0.95	0.84-1.08	0.440
Others	1.18	1.05-1.33	0.006	1.11	1.02-1.21	0.015	1.17	1.04-1.31	0.008	1.12	1.04-1.22	0.005
**T**												
T1	Reference			Reference			Reference			Reference		
T2	1.05	0.91-1.20	0.538	1.08	0.97-1.21	0.178	1.09	0.95-1.24	0.223	1.07	0.96-1.18	0.245
T3	1.29	1.11-1.50	<0.001	1.20	1.07-1.36	0.002	1.28	1.10-1.48	0.001	1.18	1.05-1.32	0.005
T4	1.55	1.34-1.78	<0.001	1.50	1.35-1.67	<0.001	1.57	1.37-1.80	<0.001	1.46	1.32-1.62	<0.001
**N**												
N0	Reference			Reference			Reference			Reference		
N1	0.98	0.88-1.10	0.764	0.95	0.87-1.03	0.176	0.95	0.86-1.06	0.361	0.91	0.85-0.99	0.022
N2	0.97	0.84-1.12	0.697	0.90	0.81-1.00	0.046	0.93	0.81-1.06	0.272	0.87	0.79-0.96	0.006
N3	1.19	1.04-1.35	0.009	0.96	0.88-1.06	0.453	1.12	0.99-1.27	0.069	0.93	0.85-1.02	0.114
**Subtype**												
HER2-positive	Reference			Reference			Reference			Reference		
Luminal A	1.58	1.23-2.03	<0.001	1.19	1.08-1.31	<0.001	1.55	1.23-1.96	<0.001	1.18	1.08-1.30	<0.001
Luminal B	1.14	0.87-1.50	0.348	0.80	0.71-0.89	<0.001	1.08	0.83-1.39	0.568	0.77	0.69-0.87	<0.001
Triple Negative	4.44	3.38-5.82	<0.001	2.63	2.37-2.93	<0.001	4.18	3.23-5.40	<0.001	2.48	2.24-2.75	<0.001
**Brain**												
No				Reference						Reference		
Yes				2.04	1.87-2.23	<0.001				1.99	1.92-2.17	<0.001
**Radiation**												
No/Unknown	Reference			Reference			Reference			Reference		
Yes	0.92	0.85-1.00	0.056	0.92	0.86-0.98	0.011	0.89	0.82-0.96	0.005	0.90	0.85-0.96	0.001
**Chemotherapy**												
No/Unknown	Reference			Reference			Reference			Reference		
Yes	0.71	0.65-0.77	<0.001	0.65	0.61-0.69	<0.001	0.67	0.62-0.73	<0.001	0.61	0.58-0.65	<0.001
**Surgery**												
BCS	Reference			Reference			Reference			Reference		
Mastectomy	1.54	1.31-1.81	<0.001	1.16	1.02-1.31	0.019	1.53	1.31-1.79	<0.001	1.14	1.02-1.28	0.027
Reconstruction	0.98	0.75-1.27	0.861	0.77	0.62-0.96	0.019	0.91	0.71-1.18	0.478	0.71	0.57-0.87	0.001
Non-surgery	2.33	2.01-2.70	<0.001	1.88	1.68-2.09	<0.001	2.34	2.03-2.69	<0.001	1.83	1.65-2.03	<0.001

### Subgroup Analyses of Metastatic Pattern

We then conducted subgroup analyses to explore selection strategies of surgical methods under different metastatic pattern circumstances. Results suggested that in bone-only metastasis pattern, in relation to BCS, patients receiving mastectomy had worse prognosis and the poorest survival belonged to non-surgery patients (BCSS: mastectomy: HR, 1.35; 95%CI, 1.15-1.60; non-surgery: HR, 2.42; 95%CI, 2.08-2.82; OS: mastectomy: HR, 1.44; 95%CI, 1.23-1.68; non-surgery: HR, 2.40; 95%CI, 2.08-2.78). Additionally, no survival difference was observed between BCS and reconstruction recipients (BCSS: HR, 1.10; 95%CI: 0.85-1.43; OS: HR, 1.11; 95%CI: 0.86-1.44). Furthermore, patients undergoing BCS had similar BCSS with mastectomy recipients together with reconstruction recipients in viscera metastasis pattern, whereas non-surgery individuals had a worse survival (mastectomy: HR, 1.04; 95%CI, 0.92-1.18; reconstruction: HR, 0.86; 95%CI, 0.69-1.06; non-surgery: HR, 1.83; 95%CI, 1.63-2.05). However, OS benefits were identified in reconstruction group compared with patients in BCS group (HR: 0.80; 95%CI: 0.65-0.99) (**Table 4**).

**Table 4:** Multivariate analysis for independent predictive factors of different metastatic patterns.

	BCSS						OS					
	Bone-only			Viscera			Bone-only			Viscera		
	HR	95%CI	P	HR	95%CI	P	HR	95%CI	P	HR	95%CI	P
**Age**												
<=40	Reference			Reference			Reference			Reference		
41-55	0.97	0.81-1.15	0.700	1.22	1.08-1.39	0.002	0.98	0.83-1.17	0.840	1.19	1.06-1.35	0.004
56-70	1.12	0.95-1.33	0.183	1.30	1.15-1.47	<0.001	1.19	1.00-1.40	0.046	1.32	1.17-1.49	<0.001
>70	1.47	1.23-1.76	<0.001	1.61	1.41-1.83	<0.001	1.67	1.40-1.98	<0.001	1.74	1.53-1.97	<0.001
**Race**												
Black	Reference			Reference			Reference			Reference		
White	0.77	0.69-0.86	<0.001	0.82	0.76-0.88	<0.001	0.75	0.68-0.84	<0.001	0.81	0.75-0.87	<0.001
Other	0.75	0.62-0.91	0.003	0.71	0.63-0.81	<0.001	0.71	0.59-0.85	<0.001	0.70	0.62-0.79	<0.001
**Marital status**												
Married	Reference			Reference			Reference			Reference		
Single	1.15	1.05-1.25	0.002	1.24	1.16-1.32	<0.001	1.18	1.09-1.29	<0.001	1.25	1.18-1.32	<0.001
**Grade**												
I-II	Reference			Reference			Reference			Reference		
III-IV	1.52	1.39-1.67	<0.001	1.39	1.30-1.49	<0.001	1.49	1.36-1.63	<0.001	1.36	1.27-1.45	<0.001
**Histology**												
IDC	Reference			Reference			Reference			Reference		
ILC	1.25	1.11-1.41	<0.001	1.09	0.95-1.25	0.209	1.24	1.10-1.39	<0.001	1.07	0.94-1.22	0.328
Others	1.16	1.03-1.31	0.014	1.09	1.00-1.19	0.041	1.15	1.02-1.29	0.019	1.11	1.02-1.20	0.017
**T**												
T1	Reference			Reference			Reference			Reference		
T2	1.08	0.93-1.24	0.307	1.11	0.99-1.24	0.073	1.12	0.98-1.28	0.088	1.10	0.99-1.22	0.078
T3	1.20	1.03-1.41	0.021	1.15	1.02-1.30	0.024	1.24	1.07-1.43	0.005	1.15	1.02-1.29	0.021
T4	1.31	1.13-1.52	<0.001	1.38	1.23-1.54	<0.001	1.38	1.20-1.59	<0.001	1.36	1.23-1.52	<0.001
**N**												
N0	Reference			Reference						Reference		
N1	1.02	0.92-1.14	0.711	0.99	0.91-1.07	0.788				0.97	0.89-1.04	0.382
N2	1.19	1.02-1.37	0.023	1.05	0.94-1.17	0.408				1.02	0.92-1.14	0.648
N3	1.37	1.20-1.58	<0.001	0.99	0.90-1.10	0.920				0.98	0.89-1.07	0.630
**Subtype**												
HER2-positive	Reference			Reference			Reference			Reference		
Luminal A	1.56	1.19-1.99	0.001	1.04	0.94-1.16	0.418	1.41	1.11-1.80	0.005	1.00	0.90-1.11	0.996
Luminal B	1.19	0.89-1.54	0.210	0.76	0.68-0.86	<0.001	1.07	0.83-1.38	0.610	0.73	0.66-0.82	<0.001
Triple Negative	4.60	3.49-6.06	<0.001	2.59	2.32-2.88	<0.001	4.03	3.11-5.22	<0.001	2.40	2.16-2.66	<0.001
**Brain**												
No				Reference						Reference		
Yes				1.91	1.73-2.10	<0.001				1.89	1.72-2.08	<0.001
**Radiation**												
No/Unknown				Reference			Reference			Reference		
Yes				0.96	0.90-1.03	0.280	1.11	1.02-1.20	0.018	0.95	0.89-1.02	0.154
**Chemotherapy**												
No/Unknown	Reference			Reference			Reference			Reference		
Yes	0.74	0.67-0.81	<0.001	0.67	0.62-0.72	<0.001	0.73	0.67-0.80	<0.001	0.64	0.60-0.69	<0.001
**Surgery**												
BCS	Reference			Reference			Reference			Reference		
Mastectomy	1.35	1.15-1.60	<0.001	1.04	0.92-1.18	0.507	1.44	1.23-1.68	<0.001	1.03	0.92-1.16	0.600
Reconstruction	1.10	0.85-1.43	0.519	0.86	0.69-1.06	0.154	1.11	0.86-1.44	0.406	0.80	0.65-0.99	0.040
Non-surgery	2.42	2.08-2.82	<0.001	1.83	1.63-2.05	<0.001	2.40	2.08-2.78	<0.001	1.76	1.58-1.96	<0.001

**Figure 3:**
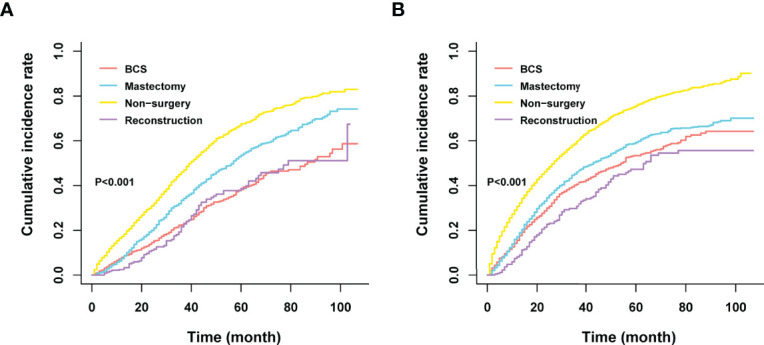
**(A)** Cumulative incidence rate of BCSD in the bone-only metastatic pattern. **(B)** Cumulative incidence rate of BCSD in the viscera metastatic pattern.

Among the 12281 patients, 7241 (58.96%) died in this retrospective study. The cumulative incidence of breast cancer-specific death (BCSD) was 53.72% (6597/12281), while that of other cause-specific death was 5.24% (644/12281). Considering the potential bias caused by competing events, competing risk model (CRM) analyses were also performed. In the univariate analysis, BCS and reconstruction recipients had a relatively lower cumulative incidence rate of BCSD than those with mastectomy and non-surgery interventions, no matter in bone-only or viscera metastatic patterns (**Figure 3**; **Table 5**). Multivariate analyses demonstrated that ten variables (age, race, marriage, grade, histology, T, N, subtype, chemotherapy, and surgery) were still independent predictive indices in the bone-only metastatic pattern while nine (age, race, marriage, grade, T, subtype, brain metastases, chemotherapy, and surgery) in the viscera metastases pattern (**Table 6**).

**Table 5:** Univariate competing risk analysis of different metastatic patterns.

	Univariate analysis (BCSD) (%)							
	Bone-only				Viscera			
	1-year	2-year	3-year	P	1-year	2-year	3-year	P
**Age**				<0.001				<0.001
<=40	6.55	15.66	26.89		13.04	30.89	41.18	
41-55	7.55	18.41	31.58		20.22	36.69	48.90	
56-70	11.71	25.11	38.37		24.86	40.80	51.95	
>70	23.39	35.02	47.25		34.81	49.37	59.60	
**Race**				<0.001				<0.001
Black	18.08	33.61	48.89		30.19	49.95	61.40	
White	12.45	23.49	35.78		24.34	39.60	50.60	
Other	8.42	22.12	34.98		19.20	32.72	44.40	
**Marriage**				<0.001				<0.001
Married	9.37	20.33	32.91		19.63	34.52	45.95	
Single	16.40	29.20	42.22		29.64	46.48	57.41	
**Grade**				<0.001				<0.001
I-II	10.69	20.19	31.76		20.55	33.88	44.97	
III-IV	16.56	32.20	46.78		28.08	45.85	57.04	
**Histology**				<0.001				0.085
IDC	12.67	24.34	36.64		24.53	40.54	51.77	
ILC	13.79	24.82	40.40		22.32	38.17	48.75	
Other	13.58	27.71	40.17		28.38	43.77	54.71	
**T**				<0.001				<0.001
T1	13.18	22.05	33.95		21.21	34.48	45.69	
T2	9.85	21.10	31.60		20.71	35.93	47.04	
T3	11.57	25.06	39.81		23.68	40.19	50.59	
T4	18.45	31.64	46.74		29.79	46.69	58.25	
**N**				0.005				0.498
N0	15.95	25.48	37.03		28.27	42.33	53.45	
N1	12.35	23.86	35.98		24.21	40.09	52.15	
N2	9.18	24.78	38.71		22.42	37.97	48.12	
N3	13.79	27.04	42.41		25.38	43.43	53.15	
**Subtype**				<0.001				<0.001
HER2-positive	13.65	22.16	29.09		21.05	34.63	44.44	
Luminal A	10.60	22.03	35.84		22.30	36.93	49.20	
Luminal B	11.00	19.20	30.46		16.15	26.35	37.50	
Triple Negative	38.17	63.25	72.77		44.01	71.07	80.21	
**Brain**								<0.001
No					22.65	38.43	49.83	
Yes					45.86	63.15	72.21	
**Radiation**				0.173				0.056
No/Unknown	13.87	25.74	38.80		25.50	41.91	52.61	
Yes	11.76	23.70	36.17		23.76	38.70	50.78	
**Chemotherapy**				<0.001				<0.001
No/Unknown	17.40	29.32	42.11		36.15	49.42	58.68	
Yes	9.76	21.63	34.36		20.28	37.30	49.24	
**Surgery**				<0.001				<0.001
BCS	7.92	14.42	22.17		15.39	29.30	39.50	
Mastectomy	7.35	19.74	32.78		17.05	34.12	45.14	
Reconstruction	2.52	11.14	21.55		8.21	22.73	30.74	
Non-surgery	17.13	30.34	44.59		30.26	46.21	57.89	

**Table 6:** Multivariate analysis of BCSD using competing risk model.

	Multivariate analysis (BCSD)					
	Bone-only			Viscera		
	HR	95%CI	P	HR	95%CI	P
**Age**						
<=40	Reference			Reference		
41-55	1.00	0.86-1.18	0.962	1.26	1.12-1.41	<0.001
56-70	1.13	0.97-1.32	0.122	1.30	1.17-1.46	
>70	1.39	1.18-1.64	<0.001	1.47	1.30-1.65	
**Race**						
Black	Reference			Reference		
White	0.81	0.73-0.91	<0.001	0.85	0.79-0.91	<0.001
Other	0.81	0.67-0.97	0.021	0.75	0.66-0.84	<0.001
**Marriage**						
Married	Reference			Reference		
Single	1.10	1.01-1.19	0.031	1.22	1.15-1.29	<0.001
**Grade**						
I-II	Reference			Reference		
III-IV	1.50	1.37-1.64	<0.001	1.35	1.27-1.44	<0.001
**Histology**						
IDC	Reference			Reference		
ILC	1.27	1.13-1.43	<0.001			
Other	1.14	1.02-1.28	0.021			
**T**						
T1	Reference			Reference		
T2	1.04	0.90-1.20	0.567	1.08	0.97-1.21	0.143
T3	1.20	1.03-1.41	0.022	1.13	1.01-1.27	0.035
T4	1.28	1.10-1.49	0.002	1.34	1.20-1.49	<0.001
**N**						
N0	Reference			Reference		
N1	1.04	0.93-1.16	0.479			
N2	1.20	1.04-1.39	0.014			
N3	1.38	1.20-1.58	<0.001			
**Subtype**						
HER2-positive	Reference			Reference		
Luminal A	1.53	1.16-2.01	0.002	1.10	0.99-1.21	0.080
Luminal B	1.21	0.90-1.61	0.205	0.81	0.72-0.91	<0.001
Triple Negative	3.99	2.96-5.36	<0.001	2.51	2.26-2.79	<0.001
**Brain**						
No				Reference		
Yes				1.78	1.62-1.95	<0.001
**Chemotherapy**						
No/Unknown	Reference			Reference		
Yes	0.77	0.70-0.84	<0.001	0.72	0.67-0.77	<0.001
**Surgery**						
BCS	Reference			Reference		
Mastectomy	1.34	1.14-1.58	<0.001	1.07	0.94-1.20	0.303
Reconstruction	1.09	0.85-1.39	0.519	0.89	0.73-1.09	0.255
Non-surgery	2.20	1.89-2.56	<0.001	1.75	1.57-1.95	<0.001

### Construction of the Nomogram Using CRM

**Figure 4:**
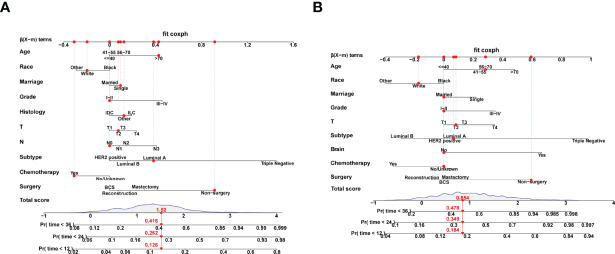
**(A)** Competing risk nomogram in the bone-only metastatic pattern. **(B)** Competing risk nomogram in the viscera pattern.

**Figure 5:**
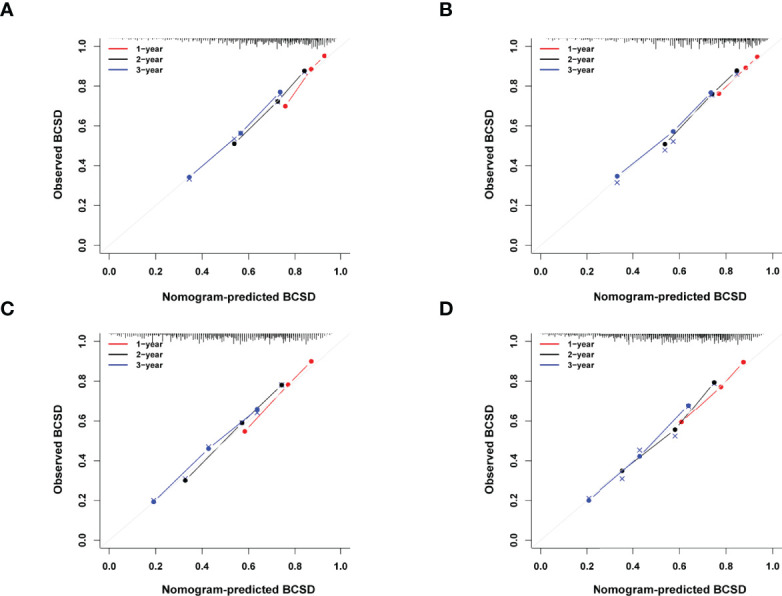
**(A)** Calibration curves for predicting BCSD at 1-, 2- and 3-year intervals in the training set in bone-only metastatic pattern. **(B)** Calibration curves for predicting BCSD at 1-,2-and 3-year intervals in the validation set in bone-only metastatic pattern. **(C)** Calibration curves for predicting BCSD at 1-, 2- and 3-year intervals in the training set in viscera metastatic pattern. **(D)** Calibration curves for predicting BCSD at 1-, 2- and 3-year intervals in the validation set in viscera metastatic pattern.

**Figure 6:**
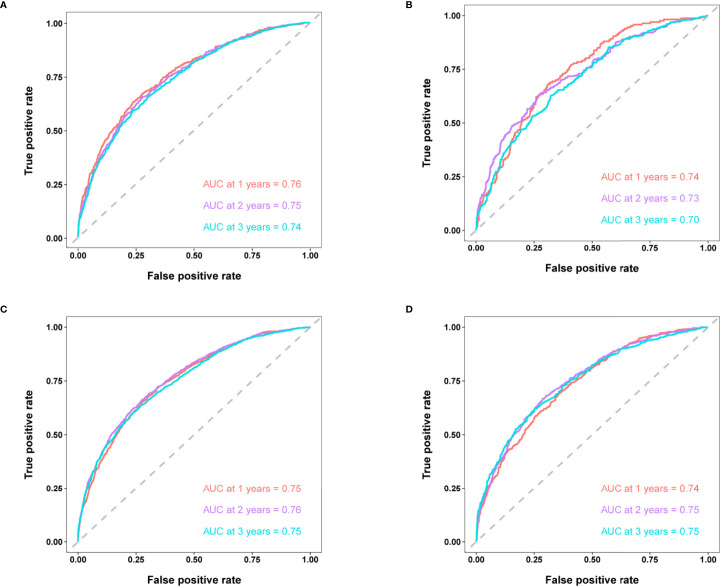
**(A)** Time-dependent ROC curves of BCSD at 1-,2- and 3-year intervals in the training set in bone-only metastatic pattern. **(B)** Time-dependent ROC curves of BCSD at 1-,2- and 3-year intervals in the validation set in bone-only metastatic pattern. **(C)** Time-dependent ROC curves of BCSD at 1-,2- and 3-year intervals in the training set in viscera metastatic pattern. **(D)** Time-dependent ROC curves of BCSD at 1-,2- and 3-year intervals in the validation set in viscera metastatic pattern.

According to the 7:3 ratio, we assigned the patients into the training and validation sets, respectively. The cutoff value was set in light of a CRM-related literature (18). Based on the screened variables, two nomograms considering metastatic patterns were developed to make precise predictions of 1-, 2- and 3-year BCSD. The probability of BCSD at these intervals can be estimated by the scale corresponding to the total score (**Figure 4**). Using the nomograms, we could prognosticate the BCSD of a given patient (bone-only metastases: 1-year=12.60%, 2-year=26.20%, and 3-year=41.60%; viscera metastases: 1-year=18.40%, 2-year=34.90%, 3-year=47.80%). Moreover, 30% of patients in the entire cohort were pitched on for internal validation. The calibration curves revealed high coherence between the nomogram-predicted and actual BCSD after one, two, and three years (**Figure 5**). The AUC values were in a range of 0.70-0.80 in both the training (bone-only metastases: 1-year=0.76, 2-year=0.75, 3-year=0.74; viscera metastases: 1-year=0.75, 2-year=0.76, 3-year=0.75) and validation sets (bone-only metastases: 1-year=0.74, 2-year=0.73, 3-year=0.70; viscera metastases: 1-year=0.74, 2-year=0.75, 3-year=0.75) (**Figure 6**).

## Discussion

Using the data from the SEER database, we constructed a Cox proportional hazard model and a competing risk model in 12281 patients diagnosed with *de novo* stage-IV BC from 2010 to 2017. Based on the variables filtered by multivariate analysis of CRM, which is widely employed in the study of oncology (19, 20), two nomograms considering metastatic patterns were constructed to predict the probability of BCSD at 1-, 2-, and 3-year intervals. As we know, this is the first large-scale SEER-based study to predict the impact of various surgical methods on survival under different metastatic patterns using competing risk analyses.

Systemic therapy is generally considered the primary treatment for patients with MBC, while locoregional therapy such as surgery is implemented to control localized symptoms such as pain and bleeding (5, 6). To date, the influence of locoregional surgery on survival has not been determined yet. In this study, Kaplan-Meier curves revealed that primary tumor resection was associated with better BCSS and OS (P<0.001). As was illustrated in our study, the median survival time of the surgery group (BCSS, 56 months; 95% CI=53–59 months; OS, 50 months; 95% CI=48–53 months) was almost 1.8-fold that of the non-surgery group (BCSS=32 months, 95% CI=31-33 months; OS=29 months, 95% CI=28–30 months). Considering potential selection bias, univariate and multivariate Cox analyses were conducted, and the results (hazard ratio, HR) summarized the risk and protective indices of survival. As shown in **Table 2**, surgery played a pivotal role in improving both BCSS and OS. Similar results were observed in several retrospective studies (7–12). The most recent research based on SEER database exhibited striking improvements in OS (before: HR, 0.57; 95%CI: 0.54-0.61; P<0.001; after: HR, 0.56; 95%CI, 0.51-0.60; P<0.001) and BCSS (before: HR, 0.56; 95%CI, 0.52-0.59; P<0.001; after: HR, 0.52; 95%CI, 0.50-0.59; P<0.001) through surgical intervention among *de novo* stage-IV patients whether before or after propensity score matching (PSM) (12). Additionally, one large-scale NCDB-based study also witnessed a survival benefit in surgery recipients with stage-IV BC. Also, OS was remarkably prolonged in the surgery group after PSM (HR, 0.68; 95%CI, 0.63-0.72; P<0.001) (11). This could be explained by the primary tumor-induced immunosuppression. The removal of lesions promoted the recovery of immunological function, preventing distant dissemination of the tumor and dislodging potential chemo-resistant cells, which led to better survival (21, 22). However, a few studies have suggested that surgical benefits were due to confounding factors caused by the design of retrospective studies (23, 24).

Despite the support from many retrospective studies, definite evidence from prospective studies is still lacking. Four prospective randomized trials observed controversial results (13–16). MF07-01 was the only trial that demonstrated survival benefits from locoregional surgery, with a remarkable improvement of 5-year OS (HR, 0.66; 95%CI, 0.49-0.88; P=0.005), while no survival advantage was found in 3-year OS (13). The ABCSG-28 POSYTIVE trial (2010–2015) and NCT00193778 (2005–2013) trial were prospective randomized trials enrolling 90 and 350 untreated patients MBC patients respectively to evaluate the impact of primary tumor surgery on OS. Patients were randomly assigned to group A (surgery following systemic treatment) and group B (systemic treatment only). Neither trial showed statistical differences in survival between the two groups (P=0.267 and P=0.790, respectively). In the former trial, the primary tumor load and lymph node metastases in group A were more serious than those in group B. In the latter, only 2% HER2 positive patients received targeted therapy, and only a minority of patients used paclitaxel during early rescue chemotherapy (14, 15). These reasons may account for the discrepancy in the outcomes. ECOG ACRIN 2018 trial revealed that primary tumor treatment notably decreased locoregional progression rate, but OS and overall quality of life were similar in patients with or without surgery (16). The results of this study aroused wide concern because only 80% of surgery recipients attained clear margins. Moreover, subgroup analyses of the metastatic patterns were not carried out.

Previous studies have indicated that biological characteristics and prognoses may vary in distinct metastatic patterns (25–27). For further analysis, we divided metastatic patterns into bone-only and viscera metastases. In our study, the survival time of bone-only metastases patients was longer than that of viscera metastases individuals, with a median BCSS of 48 months (95%CI=46-50) in the former while 33 months (95%CI=31-34) in the latter. Additionally, patients with luminal A subtype are more likely to have bone metastases (luminal A: 48.10%, luminal B: 32.74%, HER2-positive: 16.60%, triple negative: 21.18%), whereas HER2-positive and triple negative BC had a higher proportion of viscera metastasis (luminal A: 51.90%, luminal B: 67.26%, HER2-positive: 83.23%, triple negative: 78.82%), which were consistent with previous studies (28–30). To further explore the role of different surgical methods on prognosis and remove the bias from other cause-specific death, subgroup analyses of the Cox model and competing risk model regarding metastatic patterns were performed. Results demonstrated that mastectomy showed an inferior prognosis to BCS in bone-only metastases patients, whereas the worst survival belonged to non-surgery individuals. Meanwhile, no survival difference was observed between BCS and reconstruction recipients. Furthermore, patients undergoing BCS possessed similar BCSS with mastectomy and reconstruction recipients in viscera metastases pattern, while non-surgery individuals had the poorer survival. One SEER-based research had similar results, but our study was more detailed in that we subdivided mastectomy into simple mastectomy and reconstruction after mastectomy (31). We conjectured that on the premise of ensuring the safety of tumor treatments, breast reconstruction and breast-conserving surgery increase the beauty and integrity, which bring confidence and self-acceptance to patients, so as to promote the patients’ physical and psychological recovery, and consequently ameliorate survival. However, in sufferers with viscera metastases, BCS, reconstruction, and mastectomy had similar survival outcomes. We presume that due to the poor prognosis of viscera metastases patients, the survival advantages of BCS and reconstruction were attenuated.

Based on the independent predictive variables (bone-only metastases: age, race, marriage, grade, histology, T, N, subtype, chemotherapy, and surgery; viscera: age, race, marriage, grade, T, subtype, brain metastases, chemotherapy, and surgery) screened by multivariate competing risk analyses, two nomograms integrating demography, clinicopathology, and treatment information were constructed to accurately predict 1-, 2-, 3-year BCSD among stage-IV BC patients. Results of the model evaluation showed that fabulous consistency was witnessed between nomogram-predicted and actual BCSD in calibration curves. In addition, discrimination was assessed by AUC values, and the results reflected the fine sensitivity and specificity of the model. Recently, certain nomograms have been developed to predict the impact of locoregional surgery on survival among stage-IV BC patients. However, there existed a few evident limitations. For one hand, these models did not clearly discriminate different metastatic patterns, which were confirmed to be critical in survival outcomes. For another, they failed to take into account the influence of competitive events, as a result, they might overestimate the value of primary tumor surgery in stage IV BC individuals (32, 33).

This study also had several limitations that needed to be explained. First, there was no endocrine and targeted therapy in the SEER database. Second, the database lacked basic clinical data for patients, such as information on heart, lung, liver, and kidney function, as well as specific information on the metastatic focus, which might have significant impacts on treatment decisions and prognosis. Additionally, as the number of patients with *de novo* MBC was relatively small, the competitive risk model lacked effective external validation. Lastly, it was difficult to completely eliminate the bias using existing statistical methods due to the nature of retrospective cohort studies.

## Conclusion

The primary tumor surgery was associated with a lower probability of BCSD in patients with *de novo* MBC. The nomograms could offer a relatively accurate prediction of the cumulative incidence of BCSD among patients with *de novo* MBC. This will have a great significance in guiding the patients’ decisions regarding personalized precision treatment. Finally, we hope that external verification based on Chinese patients can be realized in the future.

## Data Availability Statement

The original contributions presented in the study are included in the article/**Supplementary Material**, further inquiries can be directed to the corresponding authors.

## Author Contributions

HK, ZZ, and XM designed the study and supervised the completion. HC contributed to data collection, data analysis and manuscript writing. LD, YB, and LH reviewed the background knowledge. MW, SL, and HW edited the manuscript. All authors contributed to the article and approved the submitted version.

## Funding

This study was supported by the National Natural Science Foundation of China (No. 82103129), Basic Research Program of Natural Science Foundation of Shaanxi Province (No. 2021JQ-422) (No. 2022JM-101), Prior Science and Technology Program for Overseas Chinese Talents of Shaanxi Province (No. 2020-015), and International Science and Technology Cooperation Program Project of Shaanxi Province (No. 2019KW-077) (No. 2022KW-01).

## Conflict of Interest

The authors declare that the research was conducted in the absence of any commercial or financial relationships that could be construed as a potential conflict of interest.

## Publisher’s Note

All claims expressed in this article are solely those of the authors and do not necessarily represent those of their affiliated organizations, or those of the publisher, the editors and the reviewers. Any product that may be evaluated in this article, or claim that may be made by its manufacturer, is not guaranteed or endorsed by the publisher.
